# Approaching First-Line Treatment in Patients With Advanced CMML: Hypomethylating Agents or Cytotoxic Treatment?

**DOI:** 10.3389/fonc.2021.801524

**Published:** 2021-12-13

**Authors:** Konstantinos Liapis, Ioannis Kotsianidis

**Affiliations:** Department of Hematology, Democritus University of Thrace Medical School, Alexandroupolis, Greece

**Keywords:** first-line treatment, hypomethylating agents, azacitidine (5-AzaC), decitabine (DAC), cytotoxic (or anti-neoplastic) chemotherapeutic agents, hydroxyurea (hu), chronic myelomonocytic leukemia (CMML), myelodysplastic/myeloproliferative neoplasms (MDS/MPN)

## Abstract

Chronic myelomonocytic leukemia (CMML) is a rare clonal haematological malignancy bearing characteristics of both myelodysplastic syndromes and myeloproliferative neoplasms. It primarily affects older people (median age at diagnosis ~72 years). There are many challenges encountered in its treatment. One striking issue is the lack of strong clinical evidence from large randomized clinical trials for treating this disease. Another issue is that patients with CMML have highly variable outcomes with current treatments. Additional challenges include a wider application of current knowledge, an improved understanding of pathogenesis, development of new therapies, and management of refractory cases/disease progression. It is clear that there is still progress to be made. Here, we review the available first-line treatment options for advanced CMML. Emphasis has been placed on choosing between hypomethylating agents and cytotoxic treatments, on the basis on disease-specific and patient-specific characteristics. A proper selection between these two treatments could lead to a better quality of care for patients with CMML.

## Introduction

Chronic myelomonocytic leukemia (CMML) is a mixed myelodysplastic/myeloproliferative disorder with symptoms that encompass anemia, thrombocytopenia, and splenomegaly (1). CMML has undergone several revisions in its classification reflecting the complexity of the disease. It is subdivided, on the basis of a white-cell count (WBC) of 13×10^9^/L, into dysplastic (MD) and proliferative (MP) variants (2). Outcomes of patients with MP CMML are worse than that of patients with MD CMML. The outcome is also related to the percentage of blasts in the bone marrow (BM). This value is an important part of the diagnosis and is used for a second subdivision into CMML-1 (blasts <5% in blood and <10% in BM) and CMML-2 (blasts 5-19% in blood and/or 10-19% in BM) (2).

Once diagnosed, the prime consideration is determining whether the patient needs treatment, followed by deciding the appropriate treatment. Ideally, standard management recommendations should be supported by prospective data from randomized controlled trials (RCTs). In reality, however, patients with CMML are often excluded from clinical trials (3). Thus, a major issue is the lack of strong clinical evidence from RCTs for treating CMML (4). The management of CMML, therefore, has evolved without high-quality, data-driven evidence regarding the relative benefit of different treatments.

Another issue to consider is that the outcome of treatment can be highly variable between patients, leading to divergent outcomes in different individuals (1, 4). Such variability among patients is multifactorial. Several prognostic scoring systems have been developed in recent years and the biological factors underlying this variability have been investigated (5–9). For example, the CMML-specific Prognostic Scoring System (CPSS) uses four variables (FAB and WHO CMML subtypes; erythrocyte transfusion dependence; and cytogenetic findings) to classify patents into four risk groups (low; intermediate-1; intermediate-2; and high risk) (5). Without doubt, molecular biology has changed the way we manage patients with acute myeloid leukemia (AML) and other myeloid malignancies (10–12), but in the case of CMML, full implementation of clinical genomics still has a long way to go and the molecular discoveries have not yet been translated into changes in clinical practice. The clinician who consults patients with CMML is faced with the problem of therapeutic uncertainty and biologic variation.

## When to Initiate Treatment in CMML

Not all patients need to be treated immediately. Asymptomatic patients without cytopenia or signs of myeloproliferation (e.g. palpable splenomegaly), as well as those without excess blasts (<2% blasts in peripheral-blood and <5% in BM), have a relatively stable and more indolent course and a lower probability of transformation to AML. According to the CMML treatment guidelines (published by the European Hematology Association and the European LeukemiaNet), these patients may be observed without treatment until evidence of disease progression or clinical symptoms develop (13). This is a desired approach to avoid treatment complications and the concomitant deterioration in the quality of life (QOL) in patients with asymptomatic, lower-risk CMML. However, this approach is also rooted in the principle that most treatment in CMML is largely directed towards symptom management.

Therapy should be started when CMML is symptomatic or progressive (13). More particularly, treatment is initiated for hemoglobin <10 g/dL, BM blasts >5%, platelets <100×10^9^/L, progressive leukocytosis (>30×10^9^/L), extramedullary involvement (skin lesions, pleural/pericardial effusions, lymphadenopathy), constitutional symptoms (weight loss, fever), and symptomatic splenomegaly or splenomegaly that is palpable ≥5 cm below the left costal margin (4). The key to improving the care of patients with higher risk CMML lies in providing effective therapeutic options that modify the disease course. Their management has two main objectives: to provide effective long-term disease control while maintaining patient’s QOL, and to prevent clonal evolution and transition to AML.

## Use of Hypomethylating Agents in CMML

The hypomethylating agents (HMAs) 5-azacitidine (AZA) and decitabine (DEC) induce hypomethylation by inhibiting DNA methyltransferase and are widely used in patients with myelodysplastic syndromes (MDS) and AML (14, 15). Although AZA has been approved in the USA for all patients with CMML since 2004 and DEC since 2006, the EU did not approve DEC and restricted the licence of AZA to patients with dysplastic CMML-2 in 2008 (16). Therefore, many patients with CMML in Europe do not receive treatment with HMAs and, when they do, it is often through off-label prescribing. Nonetheless, a growing body of evidence shows that HMAs may play an important role in the treatment of MP CMML (**Table 1**) (17–25). HMAs can effectively reduce leukocytosis, improve splenomegaly and extramedullary lesions. Subari and colleagues found that HMAs reduced the palpable spleen size to 50% of the baseline measurement in 45% of their patients (26). For such treatment, the overall response rate (ORR) is ~50% (30-60%) and complete response (CR) rate ~17% (10-20%). Most patients achieve a response after 3 cycles of treatment and median overall survival (OS) is ~29 months (12-37 months) (4, 13). Given that considerable time may be required for response, hydroxyurea (1g/day) may be used in patients with proliferative features during the first 3 cycles, until response is attained.

**Table 1:** Phase II studies of hypomethylating agents in chronic myelomonocytic leukemia.

Study	Number of patients	Regimen	Response rate	Median survival (months)	Progression to AML	Reference
Costa et al.	38	Azacitidine 75 mg/m^2^/day for 7 days or 100 mg/m^2^/day for 5 days every 4 weeks	ORR: 39% CR: 11% PR: 3% HI: 25%	12	NR	(17)
Adès et al.	76	Azacitidine 75 mg/m^2^ for 5-7 days every 28 days	ORR: 43% CR: 17% PR: 1% Marrow CR: 8% HI: 17%	29	31% after 1.2 years from azacitidine initiation	(18)
Wong et al.	11	Azacitidine 75 mg/m^2^ for 7 days every 28 days	ORR: 55% CR: 9% Marrow CR: 27% PR: 9% HI: 9%	17	18%	(19)
Fianchi et al.	31	Azacitidine 50-75 mg/m^2^ for 7 days in 22 patients, and 100 mg flat dose for 5-7 days in 9 patients	ORR: 54% CR: 45% PR: 3% HI: 6%	37	16% after 12.7 months	(20)
Drummond et al.	32	Azacitidine 75 mg/m^2^ for 7 days, every 28 days	ORR: 17% CR: 7% Marrow CR: 7% PR: 0% HI:3%	16	33% after 13 months	(21)
Tantravahi et al.	11	Azacitidine 75 mg/m2 for 7 days, every 28 days	ORR: 45% CR: 27% Marrow CR: 18% SD: 36% PD: 9%	30	18% at 2 years	(22)
Wijermans et al.	31	Decitabine 15 mg/m^2^ 3 times per day on 3 consecutive days, with a total dose of 135 mg/m^2^ per course, every 6 weeks	ORR: 35% CR: 10% PR: 16% HI: 19%	15	NR	(23)
Braun et al.	39	Decitabine 20 mg/m^2^ for 5 days every 28 days	ORR: 38% CR: 10% PR: 20% HI: 8%	18	NR	(24)
Santini et al.	43	Decitabine 20 mg/m^2^ for 5 days, every 28 days	ORR: 47.6% CR: 16% Marrow CR: 19% PR: 2.4% HI: 9.5%	17	57.5% after 51.5 months	(25)

ORR, overall response rate; CR, complete remission; PR, partial remission; HI, hematologic improvement; SD, stable disease; PD, progressive disease; NR, not reported.

The recent publication of the results of a large multicenter trial showed that patients with higher risk disease i.e. MP CMML, blasts ≥10%, and higher risk CMML according to the CPSS, have significantly better outcomes with HMAs compared with hydroxyurea or chemotherapy, whereas patients with lower-risk disease i.e. MD CMML with <10% blasts and lower risk CPSS, do not benefit from HMA treatment (27). Although this study provides the only direct, real-life comparison of HMAs with other available treatments, its retrospective and multicenter nature raises concerns about the presence of treatment-selection bias that cannot be overcome with Cox’s models. Also, it should be noted that the vast majority of the patients (>80%) were treated with AZA and less than 1% of patients underwent allogeneic hematopoietic-cell transplantation (HCT). Nevertheless, the study provides data that could help clinicians to address real-world questions about care options in CMML.

It is important to note that responses to HMAs are generally not durable, do not reduce mutant allele burdens, and prognosis after loss of response is dismal (median OS ~6 months) (28–30). Half the patients with primary or secondary HMA failure transform to AML (31). However, it is equally important to point out the major impact of CR on OS — that is, patients with CMML who achieve CR after HMA treatment have markedly enhanced remission duration and prolonged OS (32).

## Which Patients Are Likely to Respond to Hypomethylating Agents?

The ability to identify individuals who are unlikely to receive therapeutic benefit could facilitate a personalized approach to treatment selection and inform transplant strategies. Although clinical factors are inadequate for predicting responses to HMAs in individual patients, new molecular techniques have identified certain mutational profiles as potential biomarkers, but with conflicting results. In a retrospective series of 174 patients with CMML, patients with *TET2* mutations without *ASXL1* mutation (~25% of patients) had the highest ORR (66% versus 47% for all other genotypes) and CR rate (32% versus 11% for all other genotypes) to HMA treatment. Mutations in *ASXL1*, *RUNX1* and *CBL* were associated with low ORR and poor OS (33). By contrast, the study by Costa and co-workers indicated that *ASXL1* and *TET2* mutations did not predict response to AZA (17). Clearly, such questions can be answered only by means of dedicated, large-scale cohort studies. Several methodological barriers must be overcome to safely use mutational profiles as predictive biomarkers, as has been recently shown during the development of the new Molecular International Prognostic Scoring System (IPSS-M) for MDS (34). The identification of predictors to HMA resistance is the topic of intense research, but it must be emphasized that, in the present context, no established biomarker exists for prediction of response to HMA treatment.

## Cytotoxic Chemotherapy for CMML

Treatment should be tailored to the biology of the disease and CMML is known to be relatively resistant to cytotoxic drugs (13). Treatment regimens similar to those used to treat newly-diagnosed AML have been used in CMML. Intensive combinations of anthracycline–cytarabine, cytarabine–topotecan, or regimens including clofarabine have moderate efficacy in CMML (13, 35–37). Overall, the results of such treatments have been disappointing with a remission rate of 40%, short remission duration, and relapse rate of 90%. With the advent of HMAs, AML-type chemotherapy is used less frequently in CMML. Yet CR rates with the use of HMAs are lower (~17%) than rates achieved with induction chemotherapy (~40%). Despite major improvements in supportive care, there is a substantial risk of early death (up to 25%) and the potential for serious harm in older patients (>65 years) after induction chemotherapy, which means that the risks and benefits of treatment must be weighed carefully when formulating a treatment plan. All considered, chemotherapy remains an option for patients aged <65-70 years with minimal impairment of function who have advanced-stage or rapidly evolving disease with ≥10% BM blasts (i.e. CMML-2). The rationale behind the use of cytotoxic chemotherapy is to reduce BM blasts and aim for CR before HCT. Cytotoxic chemotherapy may also be considered for CMML with *NPM1* mutation. In particular, cases with a high *NPM1* mutational burden are associated with a higher probability of rapid transition to AML (at a median of 5 months) with myelomonocytic (M4) or monocytic (M5) differentiation and a poorer outcome, even when treated with chemotherapy (38, 39). *NPM1* mutations are uncommon in CMML occurring in <5% of cases and, if found, the alternative diagnosis of AML-M4/M5 with mutated *NPM1* should always be borne in mind (2). In contrast, the presence of *FLT3-ITD* (also occurring in <5% of cases) does not necessarily herald the onset of AML transformation (13).

## Allogeneic Hematopoietic-Cell Transplantation

This approach remains the only curative treatment of higher-risk CMML. However, it can generally be offered only to a few patients with CMML—younger patients may be offered myeloablative HCT and older patients can be offered reduced-intensity HCT from an HLA-identical donor. The conventional upper age limit for HCT is around 70 years.

Several studies have retrospectively analyzed the results of allogeneic HCT in CMML. It emerges that, at the moment, outcomes of HCT in CMML are worse than in MDS: the response rates in CMML have ranged from 17% to 50%, and treatment-related mortality (TRM) rates have ranged from 12% to 52% (40–44). The largest study of HCT to date including 513 patients found a relapse rate of 32%, non-relapse mortality (NRM) 41%, disease-free survival 27%, and OS 33% at 4 years. Disease status was the main risk factor for TRM, and the achievement of CR pre-transplant was the only predictor of survival (45).

Since the achievement of CR prior to HCT is the most important prognostic factor for a favorable outcome (45), the members of an expert panel have recommended treatment before HCT, particularly in cases with BM blasts ≥10% and/or intermediate-2 or high risk CPSS (46). In view of the lack of evidence from RCTs, the best treatment for reducing tumor burden before HCT remains a controversial issue (13, 47, 48). AZA may allow for similar outcomes after HCT as compared with induction chemotherapy (49, 50). However, the CR rate with chemotherapy is higher than HMAs (excluding patients with *TET2*+*/ASXL1*- mutations), suggesting that chemotherapy may be suitable in selected, younger patients with high blast count. Transplantation should preferably be performed early after diagnosis and after establishing the best possible response.

## Cytoreduction With Hydroxyurea

Single-agent, low-dose chemotherapy (e.g low-dose cytarabine, etoposide, 6-mercaptopurine) gives poor results in CMML (51–54). However, hydroxyurea remains an important component of CMML treatment despite its lack of disease-modifying activity. Since the classic study by Wattel and co-workers in 1996 (54), hydroxyurea is used for the control of leukocytosis, organomegaly, visceral involvement and hypercatabolic symptoms associated with advanced-stage, MP CMML. In this study, advanced-stage CMML was defined as presence of either extramedullary disease, excluding enlargement of the spleen and liver, or ≥2 of the following criteria: BM blasts >5%, neutrophils >16×10^9^/L, hemoglobin <10 g/dL, platelets <100×10^9^/L, and splenomegaly >5 cm below costal margin. The usual dose is 1 g/day (doubled in case of visceral involvement), escalated up to 3 g/day in the absence of response after 2 weeks of treatment, and adjusted to maintain WBC 4-10×10^9^/L (54). Hydroxyurea yields only partial responses, has low efficacy on visceral involvement, may lead to worsening of anemia, and survival is generally poor (median OS ~24 months) (54). Prognostic factors for lower response rates and poor OS include unfavorable karyotype (monosomy 7 or complex) and low hemoglobin level (54). In a small, retrospective series of patients with MP CMML, previous hydroxyurea exposure appeared to have a modest negative impact on the response rate to HMA treatment (55), but this finding requires prospective confirmation.

## DACOTA Trial: A Simple, Phase 3 RCT With Complex Questions

The DACOTA trial, a multicenter prospective phase 3 RCT of DEC versus hydroxyurea, included 170 patients with advanced-stage, MP CMML between October 2014 and September 2019 (84 in the DEC group; 86 in the hydroxyurea group). The data presented at ASH showed that although DEC was associated with a higher ORR as compared with hydroxyurea (56% versus 30%), no significant differences between the two groups were noted with respect to OS and event-free survival (EFS) (56). Why, then, patients that have a higher response rate with DEC do not have a superior OS or EFS? One reason might be that one third of patients with hydroxyurea subsequently received HMAs after exiting the study because of disease progression. Another possibility is that the benefit of DEC may be confined to a subset of patients with MP CMML―e.g., patients without *ASXL1* mutations and/or patients with higher risk CPSS. Or, perhaps more likely, the better disease control achieved with DEC was offset by more frequent complications and treatment delays. If so, relying on supportive care with prophylactic antimicrobial agents could be a wise decision in these cases. To put the study in perspective, the main conclusion is that, although hydroxyurea remains a valid option, HMAs, given their higher response rate, have the potential to serve as a bridge to allogeneic HCT in patients with advanced-stage, MP CMML.

## Conclusion

### First-Line Treatment in Patients With CMML: Hypomethylating Agents or Cytotoxic Treatment?

Although our understanding of the pathophysiology of CMML has improved remarkably, its treatment remains an unmet clinical need. At present, HCT is the only treatment that can induce long-term remission in CMML. Such therapy, however, is not applicable for most patients, since the median age at diagnosis is 72 years (1). Many patients have comorbidities and impaired functional status, which can lead to poor post-transplantation outcomes. In addition, the substantial risks of death and complications associated with this procedure may not justify its use in patients at lower risk. We consider HCT a treatment option for younger and fitter patients who have a poor prognosis or severe symptoms. Improving the efficacy and safety of HCT would allow many more patients to be cured.

Regardless of whether HCT is scheduled, AZA is our preferred treatment option to reduce the leukemic burden and overcome transfusion dependence in patients who need treatment, including patients with MP CMML. Yet we may consider induction chemotherapy for younger patients with adequate organ function and performance status who have advanced-stage or rapidly evolving disease with ≥10% BM blasts. Although not approved by the EU, AZA has been widely used for MP CMML for the past 10-15 years. It appears that HMAs have a disease-modifying activity in CMML, but, as is the case of MDS and AML, their effect is temporary and combinations with novel agents targeting different pathways is urgently needed in order to enhance remission duration and prolong OS (57). Current efforts in clinical research focus on the discovery of HMA combination therapies that are intended to provide an improvement in efficacy over HMA monotherapy. An important new drug in combination with HMA is venetoclax, a highly-selective, potent, oral BCL-2 inhibitor. Ongoing clinical trials are evaluating the combination of AZA with venetoclax in higher risk CMML (NCT04160052, NCT03404193). However, a recent study showed that leukemic monocytic cells often develop resistance to venetoclax due to biological properties intrinsic to monocytic differentiation, as they can shift antiapoptotic proteins from BCL-2 to MCL-1, promoting cell survival (58). Additionally, *RAS* pathway mutations, found in ~11% at diagnosis (4) but ~35% at progression/relapse (59), may confer resistance to venetoclax (57, 60). The combination of HMAs with other new molecular-targeting agents and monoclonal antibodies is also under investigation in higher-risk CMML (57).

In appropriate circumstances, cytoreduction with hydroxyurea still has a role in the care of patients with CMML. The decision for such treatment should be based on the patient’s age, symptoms, comorbidity, and disease status. In our experience, hydroxyurea should be the choice for older patients with advanced-stage, MP CMML without severe anemia, thrombocytopenia, excess marrow blasts, unfavorable cytogenetics, or higher-risk CPSS. The presence of *NPM1* mutation (~5% of patients) calls for reconsideration of the diagnosis of CMML, as AML-M4 can masquerade initially as CMML, and intensive chemotherapy could be used in younger, fitter patients or combination treatment with azacitidine plus venetoclax in older individuals (13, 60).

In approaching first-line treatment in patients with CMML, we might ask another, broader and more unsettling question: how to balance clinical practice between what is known, not known, and uncertain in our knowledge.

## Author Contributions

KL searched the literature and wrote the manuscript. IK over-reviewed the manuscript. All authors contributed to the article and approved the submitted version.

## Conflict of Interest

The authors declare that the research was conducted in the absence of any commercial or financial relationships that could be construed as a potential conflict of interest.

## Publisher’s Note

All claims expressed in this article are solely those of the authors and do not necessarily represent those of their affiliated organizations, or those of the publisher, the editors and the reviewers. Any product that may be evaluated in this article, or claim that may be made by its manufacturer, is not guaranteed or endorsed by the publisher.
